# Renal cell carcinoma: the population, real world, and cost-of-illness

**DOI:** 10.1186/s12894-022-01160-y

**Published:** 2022-12-19

**Authors:** Alessandra Buja, Giuseppe De Luca, Maura Gatti, Claudia Cozzolino, Massimo Rugge, Manuel Zorzi, Mario Gardi, Matteo Sepulcri, Davide Bimbatti, Vincenzo Baldo, Marco Maruzzo, Umberto Basso, Vittorina Zagonel

**Affiliations:** 1 Department of Cardiac, Thoracic, Vascular Sciences, and Public Health, University of Padua, Via Loredan, 18, 35131 Padua, Italy; 2 Statistics Department, University of Padua, Padua, Italy; 3 Soft-Tissue, Peritoneum and Melanoma Surgical Oncology Unit, Veneto Institute of Oncology IOV-IRCCS, Padua, Italy; 4 Department of Medicine - DIMED, Pathology and Cytopathology Unit, University of Padua, Padua, Italy; 5 Veneto Tumor Registry, Azienda Zero, Padua, Italy; 6 Unit of Urology, Ospedale Sant’Antonio, Azienda Ospedale Università-Padova, Padua, Italy; 7 Radiotherapy Unit, Veneto Institute of Oncology IOV-IRCCS, Padua, Italy; 8 Oncology 1 Unit, Department of Oncology, Veneto Institute of Oncology IOV-IRCCS, Padua, Italy

**Keywords:** Renal cell carcinoma, Cost-of-illness, Economic impact, Real-world data

## Abstract

**Background:**

The RCC treatment landscape has evolved dramatically over the past decade. The purpose of this study is to present a real-world data estimation of RCC’s cost-of-illness for this tumour’s clinical pathway.

**Methods:**

This investigation is a population-based cohort study using real-world data, which considers all RCC incident cases diagnosed in Local Unit 6 of the Province of Padua in 2016 and 2017 as registered by the Veneto Cancer Registry. Data on drug prescriptions, the use of medical devices, hospital admissions, and visits to outpatient clinics and emergency departments were collected by means of administrative databases. We evaluated the costs of all healthcare procedures performed in the 2 years of follow-up post-RCC diagnosis. The overall and annual average real-world costs per patient, both as a whole and by single item, were calculated and stratified by stage of disease at diagnosis.

**Results:**

The analysis involved a population of 148 patients with a median age of 65.8 years, 66.22% of whom were male. Two years after diagnosis, the average total costs amounted to €21,429 per patient. There is a steady increment in costs with increasing stage at diagnosis, with a total amount of €41,494 spent 2 years after diagnosis for stage IV patients, which is 2.44 times higher than the expenditure for stage I patients (€17,037). In the first year, hospitalization appeared to be the most expensive item for both early and advanced disease. In the second year, however, outpatient procedures were the main cost driver in the earlier stages, whereas anticancer drugs accounted for the highest costs in the advanced stages.

**Conclusions:**

This observational study provides real-world and valuable estimates of RCC’s cost-of-illness, which could enable policymakers to construct dynamic economic cost-effectiveness evaluation models based on real world costs’ evaluation.

## Background

Renal cell cancer (RCC) is the 9th most frequently diagnosed cancer in men, and the 14th in women, accounting for 2.90% and 2.38% of all oncological diagnoses, respectively [1]. Its incidence has more than doubled in higher-income countries over the last 50 years, and the global burden is projected to continue to increase [2]. The main kidney cancer variant is clear cell histology, accounting for more than 85% of all new cases. Papillary and chromophobe variants are less frequent [3]. The number of cases in Italy is in line with global figures [4].

The advent of increasingly sophisticated therapies, capable of improving clinical outcomes in advanced disease, have dramatically evolved the treatment landscape for RCC over the past decade, extending progression-free survival (PFS) and overall survival (OS) in certain patient populations [5–7]. Nonetheless, this disease’s high mortality and high morbidity, as well as the high cost of pharmacological therapies, make the treatment and care of RCC a major challenge for health services.

The complexity of multidisciplinary management and the need to ensure the most rational allocation of resources have led national and international agencies to develop clinical practice guidelines (CPGs) to aid clinicians in decision-making processes [5]. The objective is to guarantee equal access to personalized medicine and ensure proper resource allocation to control the system [6].

Even though different studies have demonstrated that the burden of cancer management is on the rise on a global level, the current cost components of RCC patient care have hardly been analyzed in the international literature to measure this disease’s current economic impact on healthcare systems [7]. A few recent studies have analyzed the costs of managing localized [8] and metastatic RCC (mRCC) [9–13]. The evaluation of the healthcare sustainability of actual cancer care pathways should adopt a population based perspective. In fact, data generated at the population level, taking into both early and advanced stage cases, can be useful to policymakers in determining the best resource allocation [14, 15]. Thus, this study’s objective is to perform a real-world data analysis of RCC’s cost-of-illness, taking into account the direct costs incurred for the treatment of RCC.

## Methods

### Context

The Servizio Sanitario Nazionale (SSN) is the Italian healthcare service that manages the national health service on a regional level, providing universal coverage to cancer patients completely free of charge. Its fundamental values are universality, free access, freedom of choice, pluralism in provision, and equity. Regional authorities plan and organize healthcare facilities and activities in accordance with a national health plan designed to assure an equitable provision of comprehensive care, called essential levels of assistance (Livelli Essenziali di Assistenza [LEA]), across the country. Geographically distributed Local Health Authorities (LHA) actively administer public health and community health services, as well as primary care. Hospitals provide secondary care and certain specialist treatments, while scientific institutions such as cancer centers, teaching hospitals, or accredited private providers provide tertiary and highly specialized care [16].

To ensure equitable, uniform, and effective cancer care for all residents, the Regional Authority of Veneto established the Veneto Oncology Network (Rete Oncologica Veneta [ROV]), whose mission, among others, was to set up and implement Diagnostic and Therapeutic Care Pathways (DTCPs) shared among all stakeholders, including not only clinicians but also patient advocacy groups. These pathways are clinical governance tools which, depending on the type of tumor or clinical problem, identify the best practicable pathway within the regional health organization. DTCPs are based on available scientific evidence and refer to the main international and national guidelines and recommendations. Reference is also made to Italian national and regional legislation, and to existing literature on network organization models for oncology service [6].

**Fig. 1:**
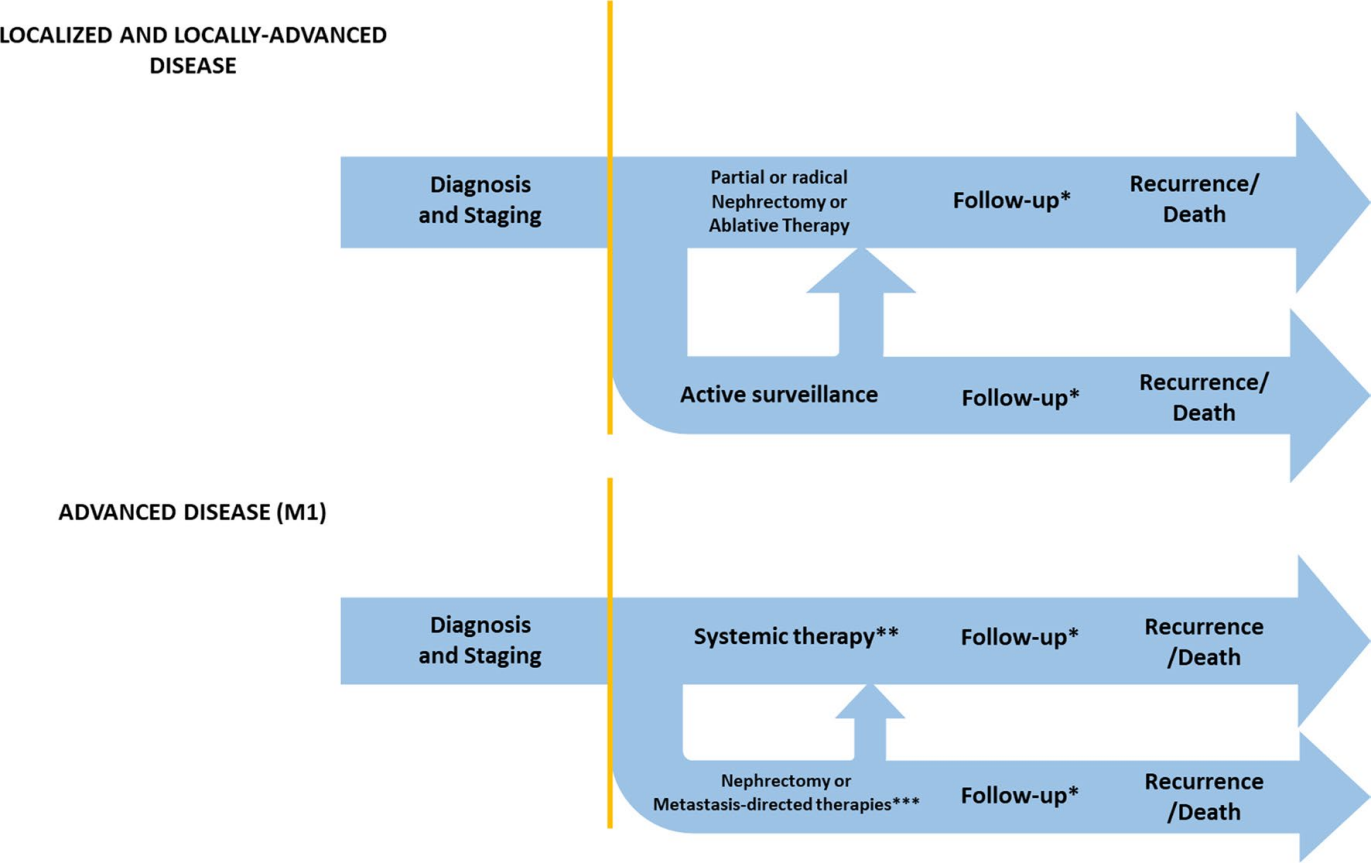
Diagnostic, therapeutic and care pathway of RCC, localized and locally advances and advanced disease. *Follow up: Low risk 6 months—abdominal ultrasonography, 12 months—Abdominal CT with contrast agent, 24 months—Abdominal ultrasonography; intermediate/high risk 6 months-Abdominal CT with contrast agent, 12 months—Abdominal CT with contrast agent, 24 months—Abdominal CT with contrast agent [18]. **Systemic therapy: first line -Sunitinib, Pazopanib, Bevacizumab + Interferon-α, Temsirolimus, Sorafenib; second line (after cytokines)—Pazopanib, Axitinib, Sorafenib, Sunitinib; second line (after VEGF/VEGFR inhibitors) – Nivolumab, Cabozantinib, Axitinib, Everolimus, Sorafenib [17]. ***Metastasis-directed therapies: metastasectomy, radiotherapy or other ablative procedures

In this context, the Veneto Oncology Network published the Diagnostic and Therapeutic Care Pathway (DTCP) for RCC, according to national [17] and international guidelines [18]. The DTCP considers every stage of the disease, from diagnosis to palliative/hospice care or follow-up, with a view to fostering coordination and sharing between hospital and territorial services/operating units involved in the care of RCC patients. Figure 1 reports an overview of the Diagnostic and Therapeutic Care Pathways for RCC, distinguishing localized and locally advanced disease from advanced disease.

### Patient data

This study is a population-based cohort study using real-world data. All RCC incident cases diagnosed in 2016 and 2017 in the area of Local Health Unit 6, Province of Padua, Northeast Italy, and recorded by the Veneto Cancer Registry were taken into consideration. The date of the first pathological diagnosis was used to define cancer incidence data. The date of the first hospital admission issuing a RCC diagnosis was used when pathologic anatomy reports were unavailable. The cases were staged according to the 8th edition of the AJCC (American Joint Committee on Cancer) classification [19], which considers patients’ medical records produced within 6 months of the date of incidence.

### Cost analysis

Data on drug prescriptions, use of medical devices, hospital admissions, outpatient and emergency room visits were taken from administrative databases (see bulleted list below).

Costs were drawn from the reimbursement tariffs established by the Veneto Regional Authority for each procedure or medical action:

- The outpatient database collects information on medical actions and procedures that can be delivered at outpatient facilities under SSN funding (e.g., radiological procedures, blood tests, pathological diagnoses, and outpatient visits) valued at the rate reported in the Nomenclatore Tariffario delle Prestazioni Ambulatoriali (NTPA), an outpatient formulary [20].
- The hospital admissions database lists the DRG (Diagnosis-Related Group) of each admission, valued at the rate reported in the Nomenclatore Tariffario delle Prestazioni Ospedaliere (NTPO), an inpatient formulary covering all hospital activities for acute or day hospital admissions [21].
- The pharmaceutical distribution databases are regional databases used to assess the costs of medical therapies (e.g., anticancer drugs) taking the doses administered into account.
- The emergency department admissions database includes the costs of each admission, derived from the rates for all medical actions and procedures performed during A&E admissions.
- The medical devices database keeps track of the costs incurred by the regional authorities to provide medical devices.

Each patient was linked via an anonymous unique identification code to all administrative data regarding their hospital admissions, outpatient care, drug prescriptions, emergency department visits, medical device usage, and hospice admissions. We considered the costs associated with 2 years of follow-up post-RCC diagnosis. The average annual real-world costs per patient, both as a whole and by single item, were calculated by weighting survival time and stratifying by stage of disease and morphology at diagnosis.

Descriptive analyses were used to analyze the cost of the sample.

## Results

A total of 148 incident cases of RCC were included in the population study: 67 cases (45.3%) were diagnosed in 2016 and 81 (54.3%) in 2017. Among these, 90 (60.81%) were stage I, 14 (9.46%) stage II, 30 (20.27%) stage III, and 14 (9.46%) stage IV; 66.22% (98) of the patients were male, and the mean age was 65.82 years (SD ± 11.3, range 39–92). As expected, clear cell histology was prevalent (63%). Other characteristics of the sample are summarized in Table 1.

**Table 1:** Sample characteristics

	Total cases N = 148
Sex n (%)	
Male	98 (66.22%)
Female	48 (32.43%)
Unknown	2 (1.35%)
Age mean (± S.D.)	65.82 (± 11.28)
Stage n (%)	
I	90 (60.81%)
II	14 (9.46%)
III	30 (20.27%)
IV	14 (9.46%)
Morphology n (%)	
Clear cell	94 (63.51%)
Chromophobe	8 (5.41%)
Non-classified/undifferentiated	2 (1.35%)
Papillary	21 (14.19%)
Unknown	23 (15.54%)
Procedure n (%)	
Radical nephrectomy	66 (44.59%)
Partial nephrectomy	76 (51.35%)
Percutaneous ablation	3 (2.03%)
None	3 (2.03%)

Table 2 illustrates that the average total costs incurred 2 years after diagnosis amounted to €21,429. Furthermore, the average per patient cost incurred during the first-year post-diagnosis amounted to €17,210, while in the second year it stood at €3934. A constant rise in costs can be observed in parallel with progression in the stage at diagnosis, with a total of €41,494 spent 2 years after stage IV diagnosis (2.44 times the amount of the management of stage I: €17.037). In particular, a rise in costs is observed as the stage progresses in the first year after diagnosis, with stages III and IV being worth 1.51 (€22,126) and 1.79 times (€26,096) the value of stage I (€14,610), respectively. In the second year, the differences in average costs between stages were more pronounced, with stage III and IV being worth 2.60 (€6320) and 6.34 (€15,397) times those of stage I (€2427), respectively. The fraction with stage III or IV disease was 34.0%, 12.5%, and 19% in patients with clear cell, papillary, or chromophobe disease, respectively. Patients with clear cell and papillary histologies had a comparable distribution of costs during the first (€17,488 and €15,331, respectively) and second years (€4222 and €3552, respectively), while those with chromophobe tumors incurred lower costs (€13,231 and €1685 in the first and second years, respectively). Patients undergoing radical nephrectomy are also characterized by significantly higher overall costs in the 2 years after diagnosis than patients who have a partial nephrectomy. (€27,804 vs. €16,315).

**Table 2:** Mean and median per patient costs (in €) at first and second year after diagnosis, stratified by stage at diagnosis, morphology and procedure

	First year				Second year*				Total*		
	n	Mean (S.D.)	Median	Mean Rate	n	Mean (S.D.)	Median	Mean Rate	Mean (S.D.)	Median	Mean Rate
Stage at diagnosis											
I	90	14,610 (5415)	13,070	–	88	2427 (3961)	1167	–	17,037 (6735)	14,237	–
II	14	14,503 (4199)	13,242	0.99	14	4891 (10,189)	1048	2.02	19,394 (11,020)	14,290	1.14
III	30	22,126 (15,052)	13,683	1.51	28	6320 (10,128)	1587	2.60	28,447 (18,343)	15,270	1.67
IV	14	26,096 (15,845)	24,688	1.79	8	15,397 (12,675)	11,864	6.34	41,494 (23,069)	36,552	2.44
Histology											
Chromophobe	8	13,231 (1002)	13,106	–	8	1685 (2306)	877	–	14,917 (2515)	13,983	–
Clear cell	94	17,488 (10,577)	13,293	1.32	91	4222 (7087)	1700	2.51	21,709 (12,796)	14,993	1.45
Non-classified/undifferentiated	2	29,626 (1007)	29,626	2.24	0	–	–	–	29,626 (1007)	29,626	1.99
Papillary	21	15,331 (8260)	13,350	1.16	20	3552 (6774)	1167	2.11	18,882 (10,790)	14,517	1.27
Unknown	23	18,095 (11,881)	13,571	1.37	19	5976 (12,039)	1043	3.55	24,070 (17,793)	14,614	1.61
Procedures											
Radical nephrectomy	66	21,394 (13,661)	1198	1.52	58	6410 (9802)	1898	2.85	27,804 (10,591)	16,007	1.70
Partial nephrectomy	76	14,066 (3220)	13,013	–	76	2249 (3802)	1144	–	16,315 (3820)	14,157	–
Percutaneous ablation	3	17,885 (7923)	12,623	1.27	3	12,935 (17,499)	591	5.75	30,820 (18,087)	13,215	1.89
None	3	4127 (2779)	4238	0.29	1	730 (0)	730	0.30	4857 (1604)	4968	0.30
Total	148	17,210 (10,312)	13,335		138	3934 (7609)	1145		21,429 (13,102)	14,606	

*Weighted by survival time

Table 3 illustrates the mean and median per patient costs by item stratified by stage. In the first year, hospitalization appeared to be the most expensive item in both early and advanced disease; in fact, it accounted for 85% and 90% of total costs in stages I and II, dropping to 70% and 50% in stages III and IV, respectively. Instead, the mean hospitalization per patient itemized costs appeared to be significantly lower in the second year. On the other hand, the average costs of hospital-prescribed drugs showed a greater impact in the later stages, rising from 17% in stage III to 36% in stage IV during the first year. However, in the second-year post-diagnosis, these costs represented the major sources of costs in stage IV (71%). Moreover, in the second-year post-diagnosis, outpatient procedures represented the first most expensive item in stage I, accounting for 55% of total costs.

**Table 3:** Specific per patient costs (in €) at first and second year after diagnosis, stratified by stage at diagnosis

Stage	Hospitalization			Outpatient visits			Emergency room			Hospital-prescribed drugs			Medical devices			Other drugs		
	Mean (s.d.)	Median	% Mean total cost	Mean (s.d.)	Median	% Mean total cost	Mean (s.d.)	Median	% Mean total cost	Mean (s.d.)	Median	% Mean total cost	Mean (s.d.)	Median	% Mean total cost	Mean (s.d.)	Median	% Mean total cost
First year																		
I	12,479 (3337)	11,615	85.46	1460 (3367)	974	10.00	99 (212)	0	0.68	270 (1457)	31	1.85	5 (43)	0	0.03	289 (384)	162	1.98
II	13,000 (3869)	11,615	89.64	859 (517)	679	5.92	104 (205)	0	0.72	257 (476)	42	1.77	7 (27)	0	0.05	276 (349)	158	1.90
III	15,549 (8045)	11,615	70.43	1825 (1717)	1267	8.27	110 (232)	0	0.50	3710 (10,183)	39	16.80	546 (1955)	0	2.47	337 (658)	76	1.53
IV	12,933 (6428)	11,615	49.56	2020 (1189)	2052	7.74	313 (414)	176	1.20	9356 (12,901)	416	35.85	764 (2192)	0	2.93	711 (668)	602	2.72
Second year*																		
I	415 (1633)	0	17.10	1324 (2349)	708	54.55	67 (173)	0	2.76	336 (2035)	0	13.84	17 (133)	0	0.70	268 (357)	154	11.04
II	1525 (3992)	0	31.19	803 (809)	722	16.42	29 (51)	0	0.59	154 (248)	13	3.15	2058 (7422)	0	42.09	321 (336)	214	6.56
III	803 (2752)	0	14.00	2053 (4066)	987	35.80	160 (293)	0	2.79	2249 (6196)	0	39.22	226 (1115)	0	3.94	243 (337)	129	4.24
IV	1190 (1598)	0	7.99	1610 (1245)	1366	10.81	335 (378)	192	2.25	10,626 (12,900)	4535	71.32	748 (1450)	0	5.02	390 (307)	406	2.62

*Weighted by survival time

## Discussion

The purpose of this study was to estimate the cost-of-illness for the management of RCC in a real-world population of 148 consecutive patients diagnosed with this tumor. This study revealed that the average cost of managing localized RCC is strongly correlated to stage at diagnosis (direct costs doubled in stage IV compared to stage I): the main cost driver for each stage in the first year was hospitalization stay; outpatient procedures were the main cost driver for earlier stages in the second year; and hospital-delivered drugs were the main cost driver for advanced stages.

The few patients with chromophobe histology incurred less costs than those with clear cell or papillary histologies. This is probably due to a smaller proportion of stages III or IV diseases (12.5%) compared to patients with clear cell subtypes (34.0%) or papillary subtypes (19%).

Few European studies focusing solely on the economic impact of managing mRCC patients have attempted to estimate the overall healthcare cost of these patients. Maroun et al. [11] led a cohort study that reported the costs of disease management and which, although significantly higher than those reported in our study (€5546 vs. €2572 per patient per month), are consistently driven by oral targeted therapies (53% of total costs vs. 62% observed in our study). Similarly, research by Cholley et al. [12] aimed to identify the explanatory factors of mRCC cost-of-illness, considering direct costs from the start of metastatic first-line treatment until death from any cause or until the last follow-up for survivors (lifetime horizon). This study demonstrated that the highest cost driver was anticancer treatment, followed by hospital stays, as confirmed by our study [12]. Comparable findings were presented by a recent study conducted in Germany in 2020, which highlighted that outpatient pharmacy expenditures in mRCC management accounted for over half the costs (€1966), followed by inpatient costs (€1205) [13]. A handful of other studies focused on estimating the average cost of cytoreductive surgery for mRCC, with Takagi et al. [9] reporting a total median cost ranging from $14,539 to $18,682 for stages I–IV, respectively.

In addition, patients undergoing radical nephrectomy presented higher costs than those treated with partial nephrectomy, due to differences in the prognosis of patients eligible for the different surgical procedures (and consequently the different medical treatments and follow-up examinations and diagnostic investigations).

To the best of our knowledge, no other recent European study has explored the cost-of-illness of RCC care from the time of diagnosis to therapy. However, a British study published in 2018 by Camp et al. provided some insight into the costs incurred by the NHS over the first year after partial nephrectomy, demonstrating that hospitalization, followed by outpatient visits, are the primary costs [11].

One limitation of this study is that it only considers the health direct cost of RCC care, sustained by the Italian healthcare service and, therefore, disregarding the out-of-pocket costs or costs of drugs covered by the trial's sponsor and the indirect costs and, therefore, preventing a social perspective analysis of the cost-of-illness.

Another limitation is the relatively small size of the sample analyzed; however, this is a population-based cohort and not center-specific, enabling an unbiased estimation of direct costs for this cancer at the population level.

## Conclusions

In conclusion, this observational study provides a real-world and valuable estimation of RCC’s cost-of-illness in Italy. This data could enable policymakers to construct dynamic economic cost-effectiveness evaluation models based on real world costs’ evaluation.

## Data Availability

The datasets generated and/or analysed during the current study are not publicly available but are available from the corresponding author on reasonable request.
